# Assessment of the dual role of *Lyonia ovalifolia* (Wall.) Drude in inhibiting AGEs and enhancing GLUT4 translocation through LC-ESI-QTOF-MS/MS determination and *in silico* studies

**DOI:** 10.3389/fphar.2023.1073327

**Published:** 2023-03-27

**Authors:** Neha Sahu, Nitesh Singh, Kamal Ram Arya, Sabbu Sathish Reddy, Amit Kumar Rai, Vijaya Shukla, Jyotsana Pandey, Tadigoppula Narender, Akhilesh Kumar Tamrakar, Brijesh Kumar, Bikash Kumar Rajak, Sumira Malik, Sarvesh Rustagi

**Affiliations:** ^1^ Department of Botany, University of Lucknow, Lucknow, India; ^2^ Botany Division, CSIR-Central Drug Research Institute, Lucknow, India; ^3^ Department of Plant Pathology, Faculty of Agriculture Sciences, SGT University, Gurugram, India; ^4^ Medicinal and Process Chemistry, CSIR-Central Drug Research Institute, Lucknow, India; ^5^ Biochemistry Division, CSIR-Central Drug Research Institute, Lucknow, India; ^6^ Sophisticated Analytical Instrument Facility, CSIR-Central Drug Research Institute, Lucknow, India; ^7^ Department of Bioinformatics, Central University of South Bihar, Gaya, India; ^8^ Amity Institute of Biotechnology, Amity University, Ranchi, Jharkhand, India; ^9^ Department of Food Technology, School of Applied and Life sciences, Uttaranchal University, Dehradun, Uttarakhand, India

**Keywords:** Bioactivity guided fractionation, GLUT4 translocation, *Lyonia ovalifolia*, protein glycation, Ericaceae, flavonoids

## Abstract

**Introduction:** Diabetes mellitus (DM) is a metabolic disorder that results in glucose accumulation in the blood, accompanied by the production of advanced glycation end products (AGEs) through glycation of cellular proteins. These AGEs interfere with insulin signaling and prevent GLUT4 membrane translocation, thereby promoting the accumulation of more glucose in the blood and causing post-diabetic complications.

**Methods:** In this study, we examine the anti-diabetic potential of *Lyonia ovalifolia* (Wall.) Drude, a well-known ethnomedicinal plant of the Indian Himalayas. Considering its various medicinal properties, we analyzed its ethanolic extract and various solvent fractions for *in vitro* antiglycation activity and antidiabetic potential, i.e., stimulation of GLUT4 translocation.

**Result and Discussions:** The results showed that the extract and fractions exhibited increased antiglycation activity and an increased level of GLUT4 translocation. Analysis of a further 12 bioactive compounds of ethanolic extract, identified through LC-ESI-QTOF-MS/MS, revealed the presence of three new compounds: leucothol B, rhodoterpenoids A, and leucothol A. Moreover, we performed molecular docking of identified compounds against key proteins of diabetes mellitus: the sirtuin family of NAD (+)-dependent protein deacetylases 6 (SIRT6), aldose reductase (AR), and tyrosine kinase (TK). The results showed that flavonoid luteolin showed the best binding affinity ((−12.3 kcal/mol), followed by eriodictyol, astilbin, and syringaresinol. An ADMET study showed that luteolin, eriodictyol, astilbin, and syringaresinol may be promising drug candidates belonging to the flavonoid class of compounds, with no harmful effects and complying with all the drug-likeness guidelines. Furthermore, molecular dynamics (MD) simulations on a 50 ns timescale revealed that AR protein was most stable with luteolin throughout the simulation period. Therefore, this study reveals for the first time that *L. ovalifolia* plays an important role in insulin homeostasis, as shown in *in vitro* and *in silico* studies.

## Introduction

Globally, diabetes mellitus (DM) or hyperglycemia is one of the most common metabolic disorders in both developed and developing nations. According to the International Diabetes Federation (IDF), diabetes affected 415 million people in 2015, and this number is expected to increase to 552 million by 2040 (Zimmet et al., 2016). A high blood glucose level leads to the accumulation of reducing sugars in the blood and the development of advanced glycation end products (AGEs) through non-enzymatic glycation of plasma proteins such as hemoglobin, serum albumin, and transferrin. In addition to altering and modifying other proteins, AGEs contribute to oxidative stress in the body by generating reactive oxygen species (Dariya and Nagaraju, 2020; Khalid et al., 2022), ultimately leading to several long-term complications of diabetes (Brownlee, 2001; Ahmed, 2005; Xu et al., 2018). These complications are considered a major concern after the onset of diabetes. Therefore, inhibition of AGEs may be one of the important prerequisites for preventing the post-translational modification of proteins (Gill et al., 2019). The most prevalent form of diabetes, type 2, is associated with a major pathophysiological condition termed insulin resistance, in which insulin-sensitive tissues (skeletal muscle, adipose tissue, and liver tissue) fail to respond adequately to the physiological concentration of insulin, altering glucose homeostasis in the process (Goldstein, 2002; Garneau and Aguer, 2019). Skeletal muscle has a major role in postprandial glucose disposal and accounts for more than 80% of insulin-dependent glucose disposal in humans (Vincent et al., 2004; Chadt and Al-Hasani, 2020).

**FIGURE 1:**
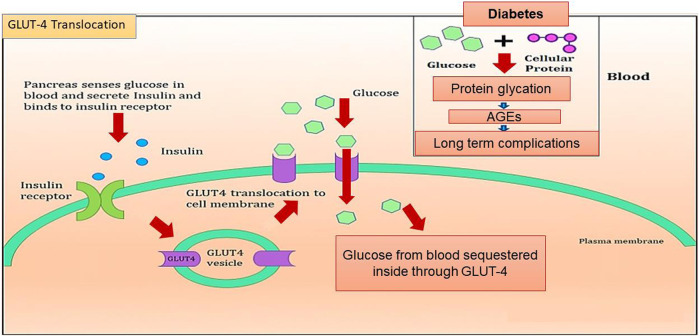
Graphical representation of GLUT4 translocation and AGE production in the cell.

**FIGURE 2:**
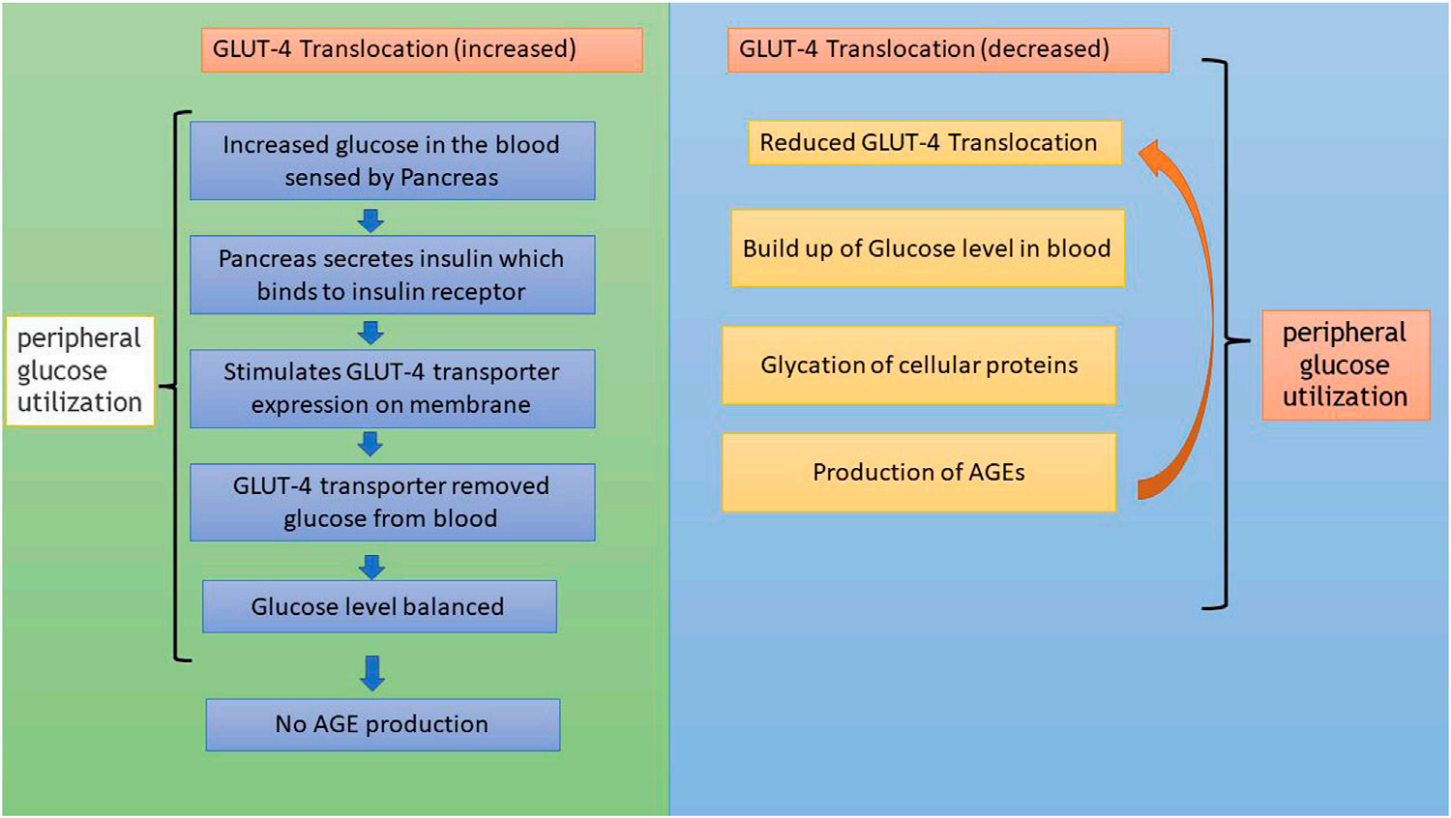
Flowchart showing GLUT4 translocation and its relationship to AGE formation.

In addition to AGE levels, GLUT4 surface translocation is also an important measure of antidiabetic activity. Insulin promotes a major signaling pathway—namely, the phosphatidyl inositol-3-kinase (PI-3-K)/protein kinase B (AKT) pathway—which stimulates the translocation and distribution of GLUT4 at the cell surface (Mackenzie and Elliott, 2014). AKT is a serine/threonine kinase that mediates most of the PI-3-K-mediated metabolic actions of insulin through the phosphorylation of several substrates, including other kinases, signaling proteins, and transcription factors. In skeletal muscle, the rate-limiting step for glucose utilization is the uptake of glucose by the cells. Glucose uptake is facilitated inside the cells by the translocation and redistribution of glucose transporter type 4 (GLUT4) from intracellular vesicles to the plasma membrane (Vincent et al., 2004). Upon insulin stimulation, the insulin receptor is phosphorylated, leading to the activation of the insulin signal transduction pathway that upregulates glucose uptake in the cells by stimulation of the translocation of GLUT4 transporters to the plasma membrane. This peripheral glucose utilization is reduced in diabetic individuals due to a reduction in GLUT4 translocation, which leads to secondary complications such as increased AGE production (Figure 1) (Klip et al., 1990; Scheepers et al., 2004). Reports also suggest that AGEs interfere with insulin signaling and prevent GLUT4 membrane translocation, promoting the accumulation of more glucose in the blood (Diamanti-Kandarakis et al., 2016) (Figure 2). Research has revealed that aminoguanidine (AG) is an effective antiglycation agent at higher doses, but cannot be recommended due to its side effects (Sadowska-Bartosz et al., 2014). Thus, there is an unmet need for new herbal antiglycation and anti-diabetic agents that not only prevent the formation of AGEs and development of insulin resistance but are also safer to use and relatively cost-effective. Estimates indicate that approximately 80% of the world’s population relies on the use of herbal medicines, which thus have better prospects on the global market (Shakya, 2016).

Today, it is widely acknowledged that medicinal plants play a significant role in conventional healthcare operations, in directing research toward new areas, and in safeguarding biodiversity (Thakur et al., 2022; Singh et al., 2022)*.* The plant *Lyonia ovalifolia*, locally known as “Angyar”, belongs to the Ericaceae family and is distributed in the mountain zones of India, Nepal, China, Japan, Cambodia, Myanmar, Thailand, Vietnam, and Malaysia. The leaves and stems of this plant have traditionally been used to treat pimples, boils, cuts, and wounds (Shah and Joshi, 1971; Bhandari et al., 2020), and have been investigated for their natural insecticide, antioxidant, antimicrobial, and immunomodulatory properties (Acharya, 2015; Sahu and Arya, 2017). Various compounds, such as grayanane diterpenoids (lyonin A-C, lyoniol D, grayanane, lyoniol-A, secorhodomollolides A and D), triterpenoids and triterpenoid glycosides (Hebecarposides A−K), iridoids (lyonofolins A-C, gelsemiol), flavonoids (quercetin 3-galactoside, apigenin, luteolin, quercetin, epicatechin, eriodictyol), lignans (lyoniside, lyoniresinol, ovafolinins A-E), and benzodioxane lignans (cupressoside A), have been isolated from the leaves and stem of this plant (Lv et al., 2016; Sahu and Arya, 2017; Yang et al., 2017; Teng et al., 2018; Zhao et al., 2018; Zhang et al., 2020). These compounds exhibit diverse biological activities. However, the bioactive compounds of *L. ovalifolia* with antidiabetic potential have been little studied, although the very small number of studies that have been conducted have revealed the presence of compounds with potential hyperglycemic properties, such as iridoids (lyonofolins A-C, gelsemiol) and flavonoids (eriodictyol), in the aerial parts of *L. ovalifolia* (Hameed et al., 2018; Hussain et al., 2018). However, the plant has yet not been explored for its targeted antiglycation and GLUT4 translocation properties. Therefore, there is a need for extensive research on this plant in terms of antidiabetic and antiglycation activity. Hence, this study evaluated the antiglycation activity of the ethanolic extract and solvent fractions of *L. ovalifolia* in a stepwise manner. In addition, extracts and bioactive fractions were examined for their role in anti-diabetic mechanisms, such as the rate of surface GLUT4 translocation in skeletal muscles. The LC-ESI-QTOF-MS/MS technique was used to identify bioactive phytochemical constituents. Furthermore, to elucidate the binding interaction of bioactive compounds, docking against the diabetes receptors aldose reductase (AR) (PDB ID: 1USO), tyrosine kinase (TK) (PDB ID: 1IR3), and SIRT6 (PDB ID: 3K35) was carried out.

## Materials and methods

### General experimental conditions

For antiglycation activity, a POLAR star galaxy spectrophotometer (BMG Labtech, Australia) was used to measure AGE fluorescence. Spectral analysis of compounds present in the samples was conducted using the Agilent 1200 HPLC system coupled with an Agilent 6520 quadrupole time-of-flight (QTOF) mass spectrometer. Further molecular docking tools were used to identify the most relevant compounds, and ADMET test tools were used to identify the toxicity level of these identified compounds.

### *In vitro* glycation assay

**FIGURE 3:**
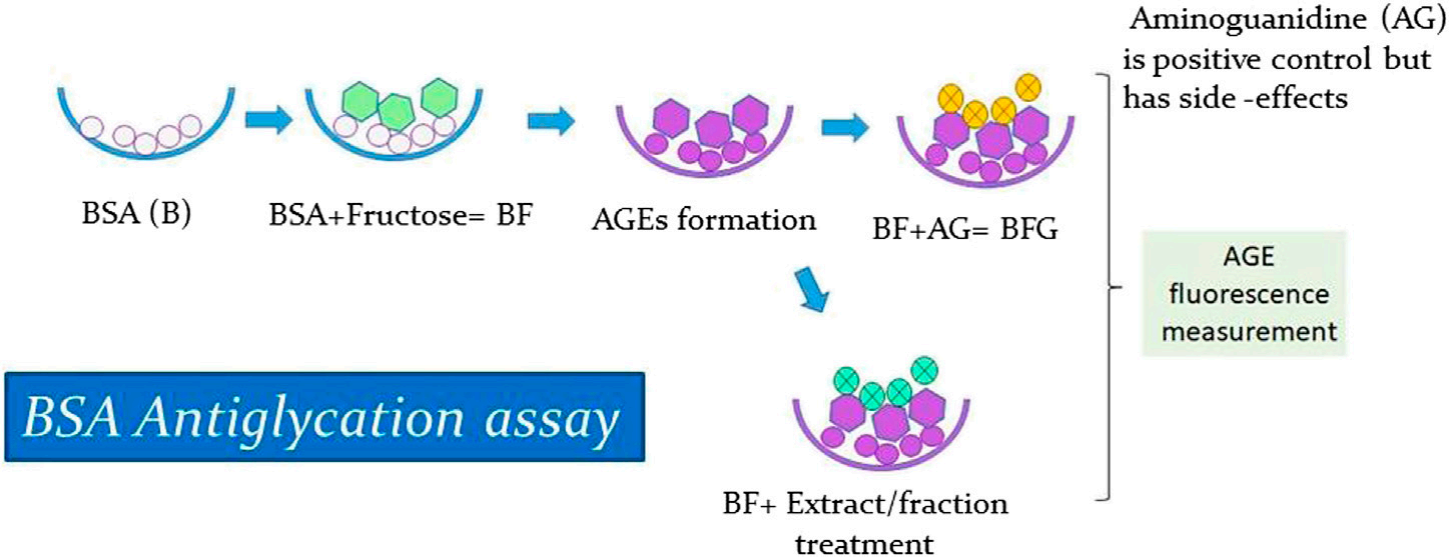
Antiglycation Assay.

*In vitro* glycation assay was conducted according to a previously published method (Sharma et al., 2002), with some minor modifications. BSA was treated with fructose to stimulate the formation of AGEs and then treated with positive control and with extract and fractions. Next, the resultant AGE fluorescence was measured to analyze the antiglycation activity of the extract and fraction (Figure 3). Since BSA shares a high homology with Human serum albumin (HSA) and is also cost-effective, it has been extensively used in *in vitro* glycation and antiglycation studies. The BSA protein sequence is particularly advantageous because it contains two tryptophan residues (Trp134 and Trp213) that are useful for spectroscopic absorption analysis, while HSA contains only one (Trp214) (Huang et al., 2004; Anguizola et al., 2013). Furthermore, amino guanidine (AG), a known inhibitor of protein glycation, was used as a positive control. The formation of AGEs was measured by fluorescence emission using a spectrofluorometer. The percentage inhibition of glycation was calculated using the following standardized formula (Sharma et al., 2002):

% Inhibition of glycation = {B − T/B} × 100

where B is the fluorescence intensity of glycated BSA (without treatment of samples) and T is the fluorescence intensity (in the presence of treated samples).

### Plant sample extraction and fractionation

**FIGURE 4:**
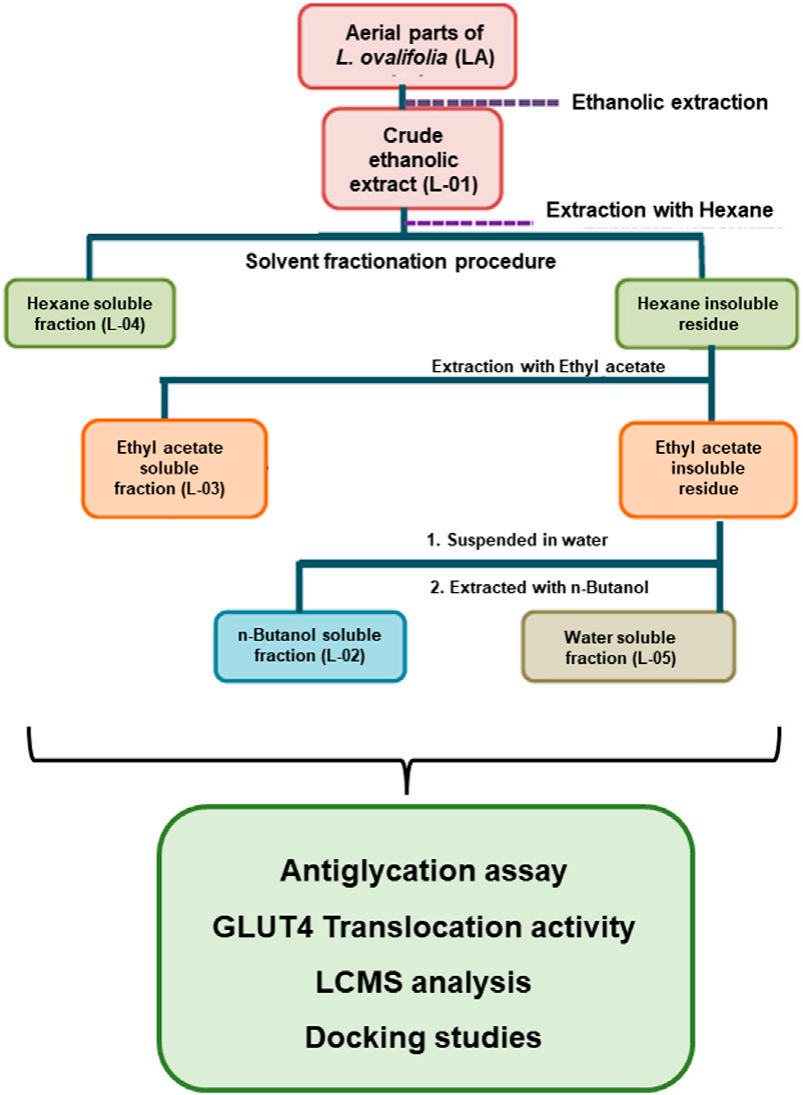
Schematic representation of bioactivity-guided fractionation of *L. ovalifolia*.

Fresh plant material of *L. ovalifolia* (Wall.) Drude was collected from Dunagiri Forest in the Almora district, Uttarakhand state, India, and identified by senior author Dr. K.R. Arya. A voucher specimen (KRA 24495) was prepared and housed in the departmental herbarium, CSIR-CDRI, Lucknow, India. Solvent extraction and fractionation (bioactivity-guided fractionation) of the aerial parts of *L. ovalifolia* were performed, as shown in Figure 4. Bioactivity-guided fractionation includes phytoextraction and fractionation of a plant sample, along with simultaneous bioactivity testing of the resultant extract and fractions. For details on the method of bioactivity-guided fractionation employed, see Huang et al. (2011a, 2011b) and Sahu et al. (2018).

Finally, the dried crude extract L-01 and fractions L-02, L-03, L-04, and L-05 thus obtained were subjected to antiglycation activity with simultaneous phytochemical analysis and GLUT4 translocation activity.

### GLUT4 translocation

In skeletal muscle, translocation and redistribution of GLUT4 to the plasma membrane is a characteristic feature in increased glucose uptake and the utilization effect of insulin. The bioactive crude extract (L-01) was analyzed for GLUT4 translocation at 10 and 25 μg/mL concentrations and compared to control (unstimulated) samples and insulin-treated samples. The effect of the extract and selected bioactive fraction on surface GLUT4 level in non-permeabilized L6-GLUT4 myc myotubes was measured via an antibody-coupled colorimetric assay. Shortly after the treatment period, the cells were washed in ice-cold PBS (154 mM NaCl, 5.6 mM Na_2_HPO_4_, 1.1 mM KH_2_PO_4_) supplemented with 1 mM CaCl_2_ and 1 mM MgCl2 (pH 7.4). The cells were then fixed in 3% paraformaldehyde for 30 min and quenched in 100 mM glycine for 10 min, all at 4°C. The cells were blocked in 5% skimmed milk for 15 min and then incubated with anti-myc antibody solution (1.0 μg/mL in PBS with 3% skimmed milk) for 1 h at 4°C. After labeling, excess antibodies were removed by extensive washing in ice-cold PBS. Cell-surface GLUT4 bound antibodies were probed by HRP-conjugated secondary antibodies, followed by the detection of bound HRP via o-phenylenediamine assay. The absorbance was read at 492 nm using a Spectromax Elisa Reader. The fraction of GLUT4 at the cell surface, measured in triplicate, was expressed as fold stimulation with respect to unstimulated control cells.

### LC-ESI-QTOF-MS/MS analysis of bioactive samples

All the samples (i.e., L-01, L-02, L-03, L-04, and L-05) were dissolved in HPLC-grade methanol and filtered through a syringe filter for LC-ESI-QTOF-MS/MS analysis. Chromatographic separation was performed on an Agilent 6520 quadrupole time-of-flight (QTOF) mass spectrometer connected to an Agilent 1200 HPLC system via a dual electrospray ionization (ESI) interface (Agilent technologies, USA). The mobile phase consisted of 0.1% formic acid aqueous solution (A) and methanol (C) with a flow rate of 0.5 mL/min under the gradient program of 35% to 90% (C) for an initial 10 min, then 90% (C) from 10 to 20 min, 90% to 35% (C) from 20 to 25 min, and 35% (C) from 25 to 30 min. The sample injection volume was 5 μL.

### Ligand–protein preparation and docking analysis

Of the 12 bioactive compounds identified from the ethanolic extract, data on ten of these were downloaded from the PubChem database in PDB format. Screening of the receptor structures was conducted using the AutoDock molecular graphics system.

### Molecular docking

In accordance with a survey of the literature, and with the crystal structures of the proteins serving as important regulators in several biosynthetic pathways of DM, we selected three human proteins: one from the sirtuin family of NAD(+)-dependent protein deacetylases, namely SIRT6 (PDB ID: 3K35), and the other two were aldose reductase (PDB ID: 1USO) and tyrosine kinase (PDB ID: 1IR3). The dimensional structures of 1IR3, 3K35, and 1USO were downloaded from the Protein Data Bank in docking PDB format. All non-protein molecules, including water and other atom locations, were eliminated using the AutoDock software. Docking of compounds into prepared proteins was then carried out using the AutoDock software to achieve accurate docking. The inhibition constant, binding energy, and ligand efficiency were subsequently calculated (Singh et al., 2021a). At the end of the docking process, the best-conforming compounds with the lowest binding affinity energy in kcal/mol were selected for further analysis. The top binding interactions obtained by molecular docking were further illustrated in terms of the residues involved in interactions in order to explain the in-depth pattern of interaction between the docked ligands and proteins.

### ADMET analysis and drug-likeness

The compounds with the best binding affinity were further evaluated in *in silico* ADMET studies. Studies of ADMET (absorption, distribution, metabolism, excretion, and toxicity) evaluate properties that are important and useful in the production of new natural compounds with improved pharmacokinetic and pharmacodynamic properties. The SwissADME, vNN process, and molsoft tools were used to study the ADMET properties of the screened substances (Singh et al., 2021b). Molinspiration, an online screening server, was used to study the pharmacodynamic properties of effective ligands.

### Molecular dynamics simulations

Molecular dynamics (MD) simulations were run for 50 ns using the GROMACS554A7 program to better understand the overall stability of the best identified non-bonding interactions between the protein and ligand (Rajak et al., 2022). MD simulation illustrates the strength of intermolecular interactions, dynamic conformational changes, patterns, and other characteristics. The best four complexes (luteolin, eriodictyol, astilbin, and syringaresinol) with aldose reductase (AR) as a binding site were selected for MD simulation. The root-mean-square deviation (RMSD), intermolecular hydrogen bond interactions, root-mean-square fluctuations (RMSF), and radius of gyration (RoG) were analyzed to elucidate the conformation, structure, and compactness of the complexes. The RMSD, RMSF, and RoG were recorded for 50 ns.

### Statistical analysis

All experiments were performed in triplicate. Data are expressed in the form mean ± standard error of the mean (SEM). The statistical significance of the results was evaluated by one-way ANOVA, using the Tukey multiple comparison test, with the help of the GraphPad Prism 7.03 software. Results were interpreted as highly significant (***) at *p* < 0.001 and (*) *p* < 0.01 and significant at (**) *p* < 0.05.

## Results

**FIGURE 5:**
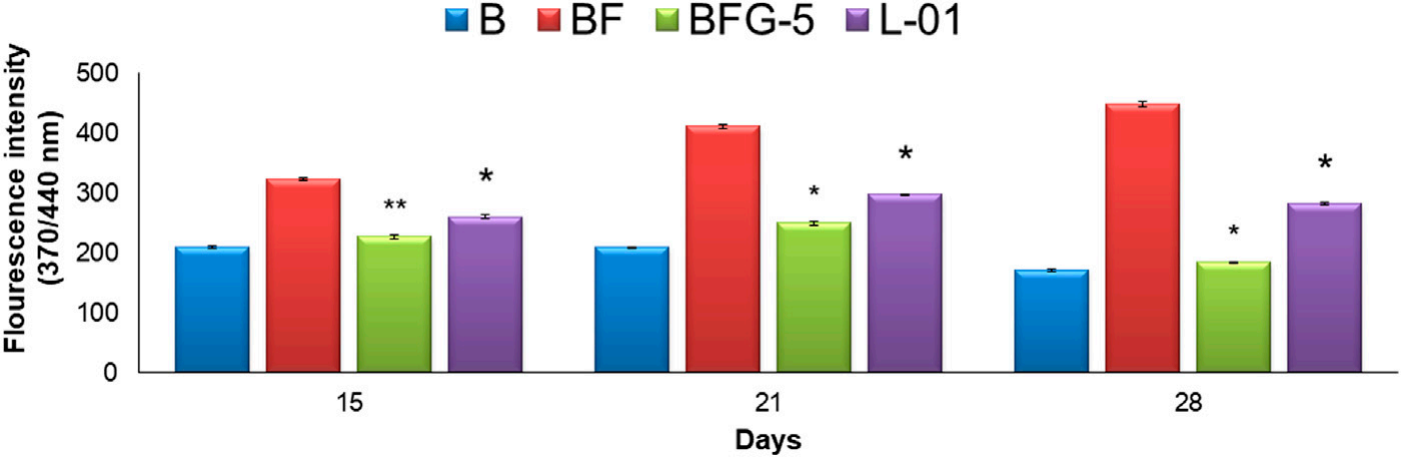
Effect of L-01 on the formation of fluorescent AGEs in BSA incubated with fructose. p* < 0.01, p** < 0.05, p*** < 0.001 relative to control i.e., compared to BSA/Fructose (BF) on same duration. (B: BSA, BF: BSA/fructose, BFG-5: AG in BSA/fructose at 5 mM, L-01 in BSA at 100 μg/mL).

**FIGURE 6:**
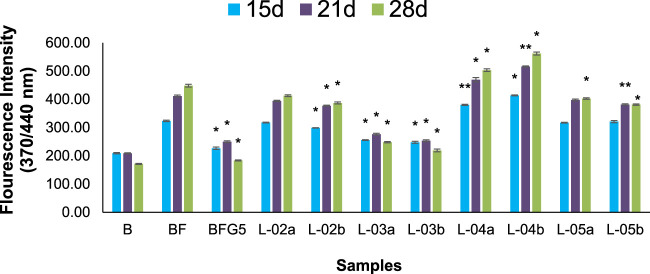
Effect of different solvent fractions of *L. ovalifolia* on the formation of fluorescent AGEs in BSA incubated with fructose. *p* *< 0.01, *p* **< 0.05, *p* ***< 0.001 relative to control i.e., compared to BSA/Fructose (BF) on same duration (L-02a, L-03a, L-04a, L-05a at 50 μg/mL and L-02b, L-03b, L-04b, L-05b at 100 μg/mL concentration); d: days.

The production of AGEs in serum albumin samples is characterized by the presence of a series of fluorescent compounds associated with strong fluorescent emission. In the present experiments, incubation of BSA (bovine serum albumin) with fructose (BF) for 28 days showed enhanced fluorescent emission, indicating increased formation of AGEs. Moreover, incubation of BF with L-01 (100 μg/mL) for 15, 21, and 28 days showed a significant (*p <* 0.01) reduction in AGE-associated fluorescent emission of 19.56%, 27.63%, and 36.95%, respectively, compared to positive control AG (59.01%) (Figure 5 and Table 1). Among the solvent fractions, L-03 at 100 μg/mL concentration (L-03b) was found to be the most effective (*p <* 0.01), with 23.45% and 38.40% AGE inhibition at 15 and 21 days, respectively, as compared to the other fractions (Figure 6 and Table 1). Remarkably, after 28 days of incubation, L-03 showed a significant increase in percent AGE inhibition (51.12%), compared to the positive control of 59.01% inhibition at 5 mM concentration (BFG5) (*p <* 0.01). The inhibitory effects of L-03 on fluorescent emission of AGEs prove its potential in inhibiting the biosynthesis of AGEs (Table 1).

**TABLE 1:** Percent inhibition of fluorescent AGEs by L-01 and L-03 (in glycated BSA) over 15–28 days of incubation.

Sample	% Glycation inhibition (days)			
	Conc. (µg/mL)	15	21	28
L-01	100	19.56 ± 0.78	27.63 ± 0.27	36.95 ± 0.78
L-03	50	20.99 ± 0.64	32.8 ± 1.34	44.58 ± 0.96
	100	23.45 ± 1.63	38.4 ± 0.85	51.12 ± 1.36
AG^a^ (mM)	5	29.97 ± 0.81	39.22 ± 0.47	59.01 ± 0.35

Values are expressed in the form mean ± SEM; n = 3 (number of samples).

L-01: ethanolic extract; L-03: ethyl acetate fraction.

^a^ Amino guanidine (positive control).

**FIGURE 7:**
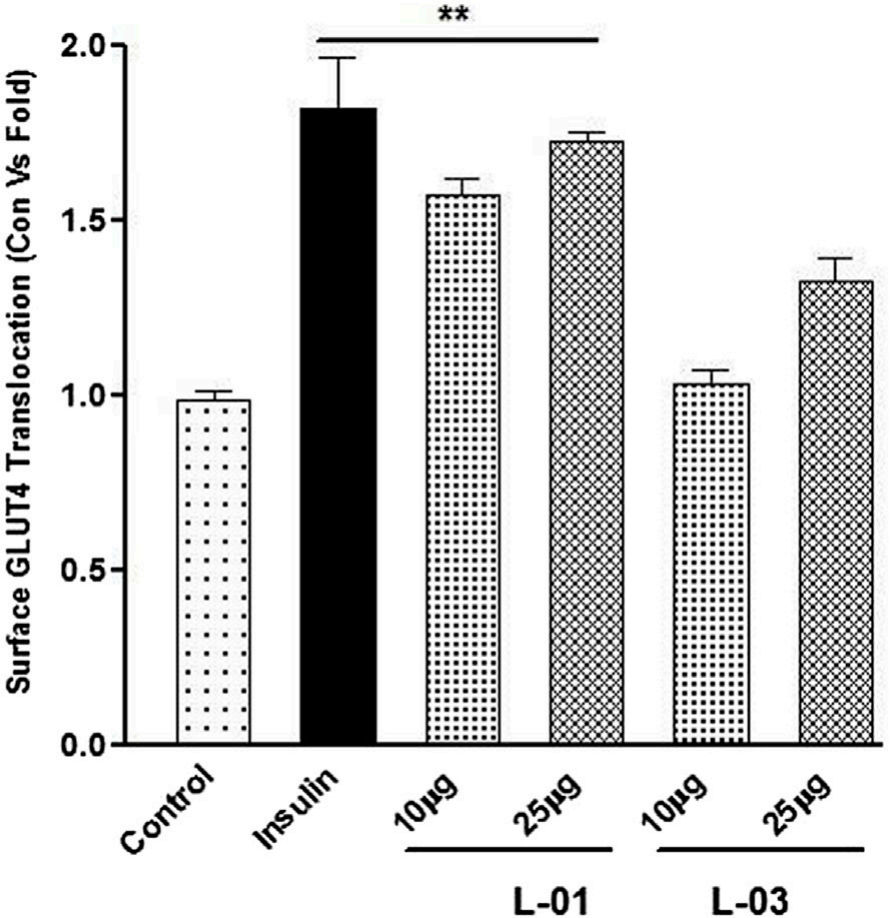
Dose-dependent effect of extract (L-01) and ethyl acetate fraction (L-03) on stimulation of GLUT4 translocation in L6 GLUT4 myc myotubes. Cells were incubated with either L-01 or bioactive fraction L-03 at 10 and 25 μg/mL concentration for 16 h, and rate of GLUT4 translocation was determined. Results shown are mean ± SE of the results of three independent experiments, each performed in triplicate. p* < 0.01, **p < 0.05, p*** < 0.001 relative to control.

**FIGURE 8:**
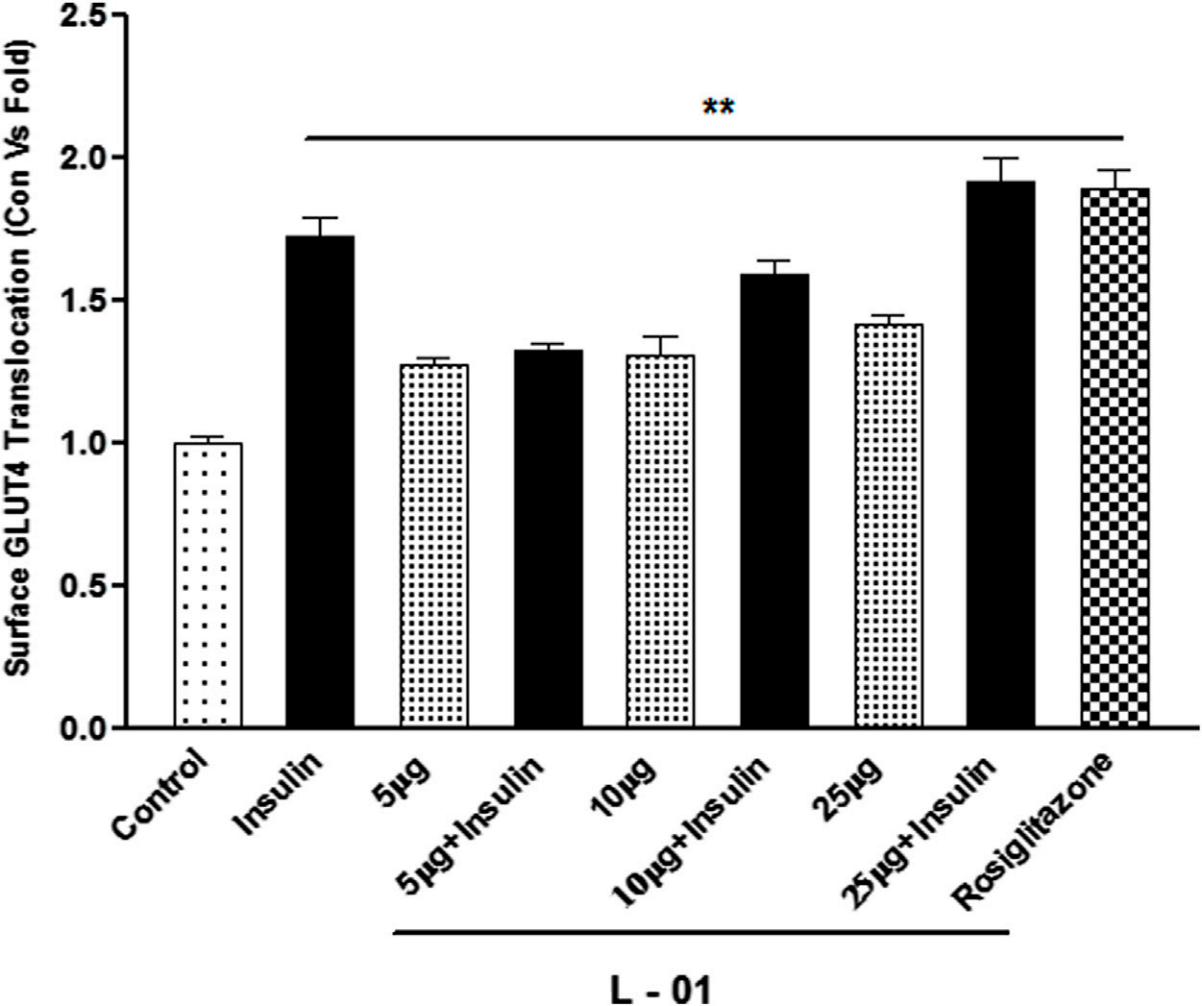
Dose-dependent effect of extract L-01 on insulin-stimulated GLUT4 translocation in L6 GLUT4 myc myotubes. Cells were incubated with extract L-01 at 5, 10, and 25 μg/mL for 16 h, followed by 3 h of serum starvation with a subset of cells stimulated with 100 nM insulin for 20 min, followed by determination of the GLUT4 translocation rate. Results shown are mean ± SE of the results of three independent experiments, each performed in triplicate. p* < 0.01, **p < 0.05, p*** < 0.001 relative to control.

GLUT4 translocation assay showed that L-01 significantly stimulated the abundance of GLUT4 molecules on the surface by up to 1.5- and 1.8-fold at 10 and 25 μg/mL concentrations, respectively, as compared to unstimulated samples (Figure 7). Moreover, the ethyl acetate fraction (L-03) also showed 1–1.2-fold GLUT4 translocation compared to unstimulated samples (Figure 7). This result meant that L-01 at 25 μg/mL was found to be most effective in GLUT4 translocation (Figure 8). The translocation rate further increased when insulin-stimulated cells were treated with L-01 (25 μg/mL), revealing the highest-fold GLUT4 translocation in a dose-dependent manner (Figure 8). This observed biological response of L-01 was comparable to the positive control rosiglitazone.

**FIGURE 9:**
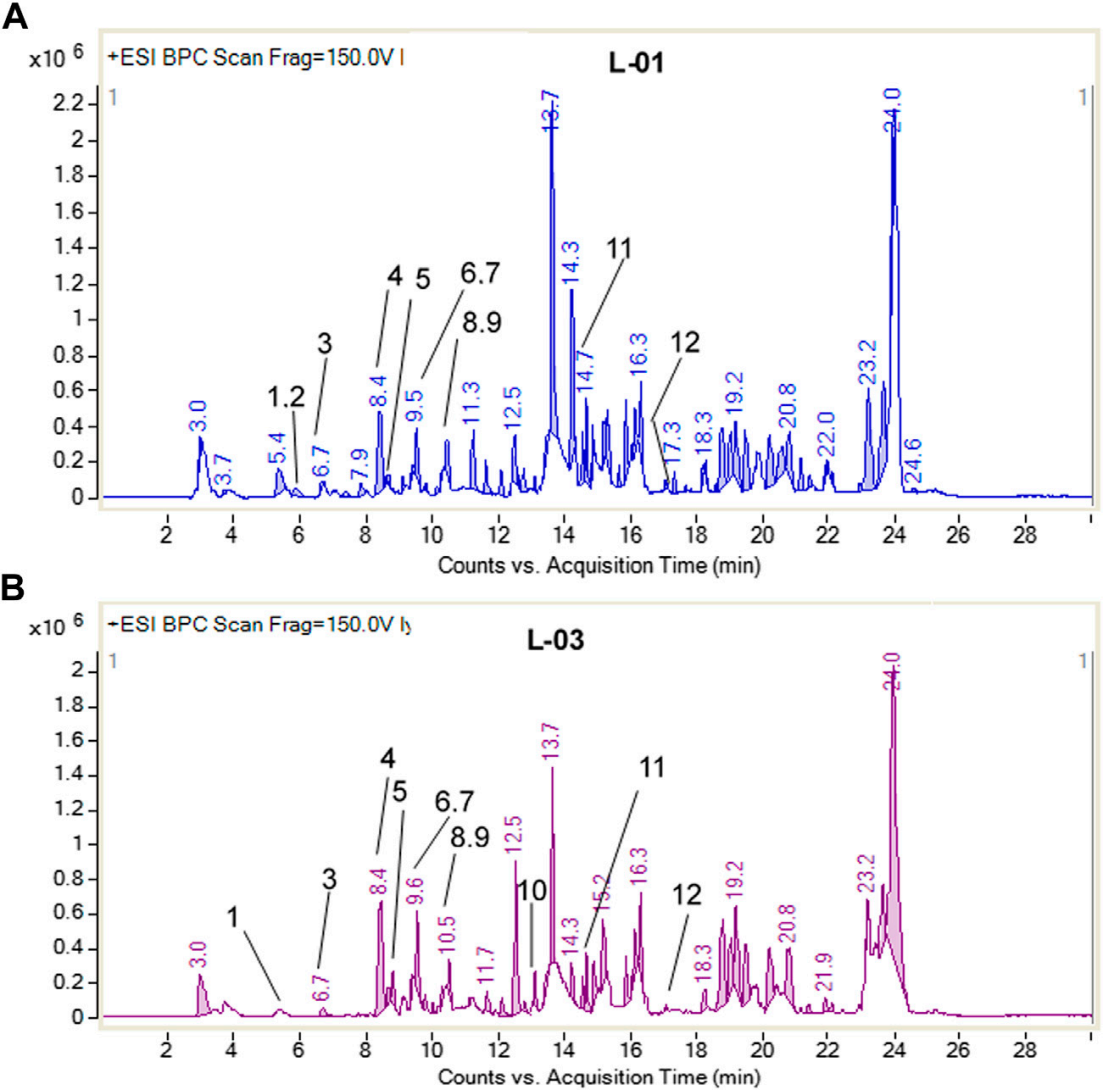
Base peak chromatogram of **(A)** L-01 and **(B)** L-03, showing 12 compounds present.

**FIGURE 10:**
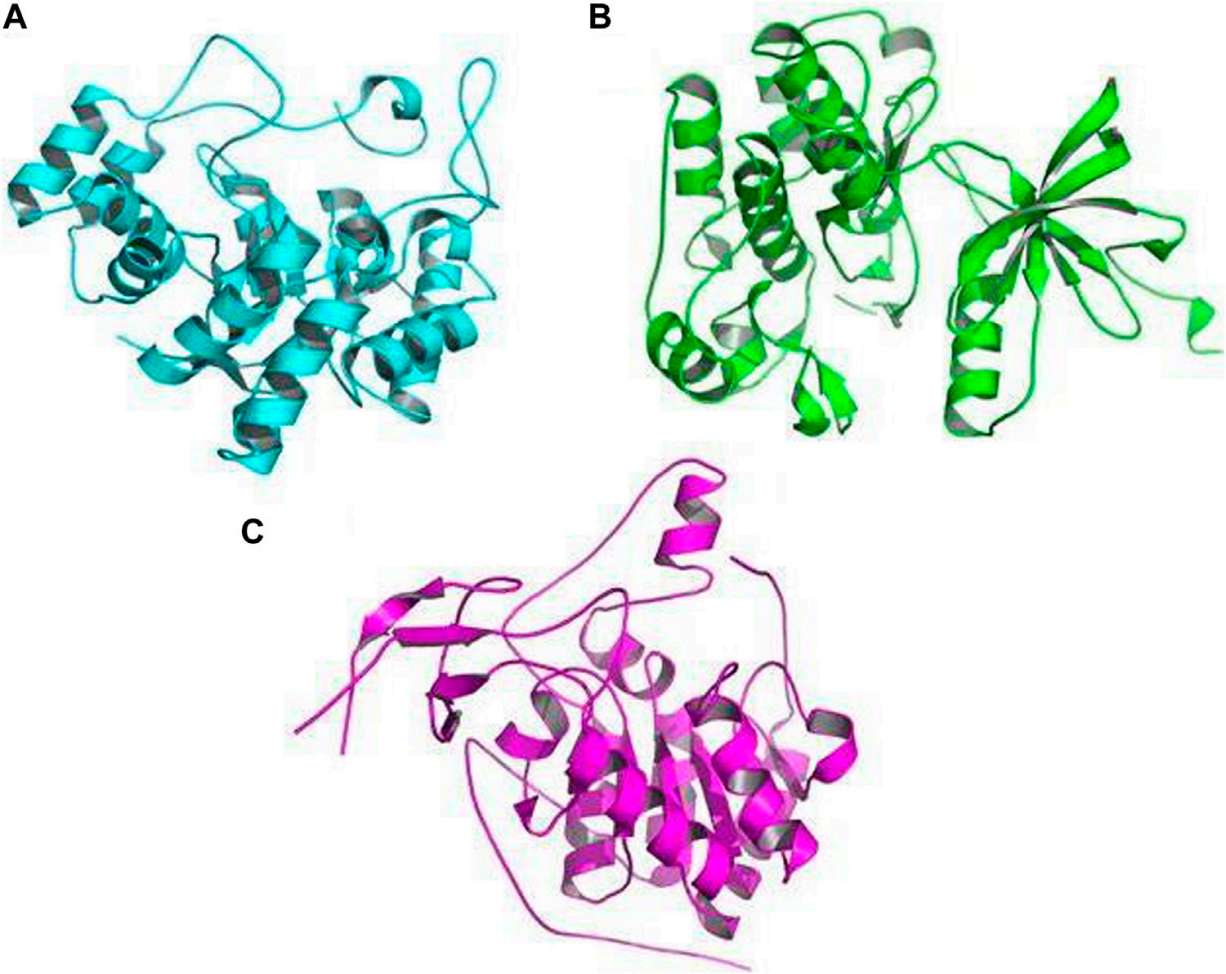
Crystal picture receptors: **(A)** aldose reductase (AR) (PDB ID: 1USO), **(B)** tyrosine kinase (TK) (PDB ID: 1IR3), and **(C)** SIRT6 (PDB ID: 3K35).

Moreover, LC-ESI-QTOF-MS/MS determination of L-01 and L-03 showed the presence of 12 major compounds belonging to the classes of lignans, flavonoids, flavonoid glycosides, and terpenoids (Table 2 and Figures 9A, B). Of these 12 major compounds, nine had been previously reported for this plant: (**1**) 5,5’dimethoxysecoisolariciresinol, (**2**) lyoniresinol, (**3**) syringaresinol, (**4**) astilbin or dihydroquercetin-3-O-rhamnoside, (**5**) quercetin 3-galactoside, (**6**) eriodictyol, (**7**) quercetin-3-O-rhamnoside or quercitrin, (**8**) luteolin, and (**9**) lyoniol C (Kashima et al., 2010; Acharya, 2015; Sahu & Arya, 2017). The remaining three compounds—(**10**) leucothol B, (**11**) rhodoterpenoids, and (**12**) leucothol A—are reported for the first time in this species. Of these 12 compounds, the three lignans (5,5’dimethoxysecoisolariciresinol, syringaresinol, and lyoniresinol) were identified and characterized by MS spectra and (RT) retention time compared with their corresponding standards analyzed under identical conditions (Table 2 and Figures 10A, B). The remaining compounds (**4–12**) were tentatively identified on the basis of their positive ESI mass spectra. Moreover, phytochemical analysis of the ethyl acetate fraction (L-03) also indicated the presence of the same 12 compounds identified in the ethanolic extract (L-01), with the exception of lyoniresinol. This indicates that most of the bioactive compounds of L-01 possess ethyl acetate solubility.

**TABLE 2:** HPLC-ESI-QTOF-MS determination of ethanolic extract (L-01) and ethyl acetate fraction (L-03) of *L. ovalifolia*.

S.N.	RT (retention time)	Error	Calculated [M+H]^+^	Observed [M+H]^+^	Molecular formula	Identified compound	L-01	L-03
1	5.9	0.71	423.2013	423.2011	C22H30O8	DLR* ^Ϯ^	+	+
2	6	0.08	421.1857	421.1859	C_22_H_28_O_8_	Lyoniresinol* ^Ϯ^	+	-
3	6.8	0.06	419.17	419.17	C_22_H_26_O_8_	Syringaresinol* ^Ϯ^	+	+
4	8.4	1.76	451.1235	451.1243	C_21_H_22_O_1 1_	Astilbin^#^	+	+
5	8.8	0.17	465.1028	465.1028	C_21_H_20_O_1 2_	Q3G^#^	+	+
6	9.4	2.82	289.0707	289.0715	C_15_H_12_O_6_	Eriodictyol^©^	+	+
7	9.6	2.36	449.0499	449.0506	C_21_H_21_O_1 1_	Q3R^#^	+	+
8	10.4	0.19	287.055	287.0551	C_15_H_10_O_6_	Luteolin^#^	+	+
9	10.7	0.91	359.2064	359.2063	C_18_H_30_O_7_	Lyoniol C	+	+
10	13.2	0.69	353.2323	353.232	C_20_H_32_O_5_	Leucothol B^** ɸ^	+	+
11	14.6	1.19	473.3625	473.3631	C_30_H_48_O_4_	Rhodoterpenoids A^† ɸ^	+	+
12	16.9	0.2	319.2268	319.2268	C_20_H_30_O_3_	Leucothol A** ^ɸ^	+	+

*Lignan ^#^Flavonoid glycoside ^©^Flavonoid **Diterpenoid ^†^Triterpenoid; + compounds present, - compounds not detected.

^ɸ^Compounds reported for the first time in *L. ovalifolia*; L-01: ethanolic extract; L-03: ethyl acetate fraction.

^Ϯ^Standard methods were used to confirm presence; DLR, 5,5′-dimethoxysecoisolariciresinol; Q3G, quercetin-3-O-galactoside; Q3R, quercetin-3-O-rhamnoside.

The receptors used for docking with bioactive compounds were aldose reductase (AR) (PDB ID: 1USO), tyrosine kinase (TK) (PDB ID: 1IR3), and SIRT6 (PDB ID: 3K35) (Figures 10A–C). After docking, it was observed that luteolin achieved the best docking score (12.3 kcal/mol) against the target receptor aldose reductase (PDB ID: 1USO). On the basis of the highest docking score and lowest binding energy, the top ligand was identified and hypothesized to be the most effective ligand against target receptor aldose reductase (PDB ID: 1USO). Among the ten ligands that were screened against the three selected receptor targets, the compounds with the best docking pose scores were predicted to be effective; their results are shown in Table 3 which presents the binding energy of ligand–receptor interactions. In our *in silico* analyses, we found that syringaresinol had a binding energy of −11.4, −7.9, and −7.4 kcal/mol against aldose reductase (AR) (PDB ID: 1USO), tyrosine kinase (TK) (PDB ID: 1IR3), and SIRT6 (PDB ID: 3K35), respectively. Astilbin had a binding energy of −11.5, −9.0, and −8.2 kcal/mol against aldose reductase (AR), SIRT6, and tyrosine kinase (TK), respectively. Lyoniol-A had a binding energy of −9.2, −6.8, and −6.7 kcal/mol against aldose reductase (AR), tyrosine kinase (TK), and SIRT6, respectively. Eriodictyol had a binding energy of −12.1, −9.1, and −8.0 kcal/mol against aldose reductase (AR), SIRT6 and tyrosine kinase (TK), respectively. Lyoniresinol had a binding energy of −9.0, −6.5, and −6.0 kcal/mol against aldose reductase (AR), SIRT6 and tyrosine kinase (TK), respectively. Luteolin had a binding energy of −12.3, −8.9, and −8.6 kcal/mol against aldose reductase (AR), SIRT6, and tyrosine kinase (TK), respectively. Quercitrin had a binding energy of −10.4, −8.9, and −8.6 kcal/mol against aldose reductase (AR), tyrosine kinase (TK), and SIRT6, respectively. Rutin had a binding energy of −10.7, −8.7, and −8.3 kcal/mol against aldose reductase (AR), tyrosine kinase (TK), and SIRT6, respectively. Leucothol A had a binding energy of −10.7, −9.1, and −7.5 kcal/mol against aldose reductase (AR), SIRT6, and tyrosine kinase (TK), respectively. Finally, leucothol B had a binding energy of −9.1, −8.3, and −7.7 kcal/mol against aldose reductase (AR), SIRT6, and tyrosine kinase (TK), respectively. As aldose reductase is reported to be involved in the rate-limiting enzyme of the polyol pathway, converting excess D-glucose to D-sorbitol with NADPH as a co-factor, it is crucial in the treatment of diabetic microvascular problems. Aldose reductase is also involved in lipid metabolism. One of the best known sirtuins in mammals, SIRT6 is involved in a number of cellular functions, including DNA repair, maintenance of glucose balance, and longevity. SIRT6 is a NAD+-dependent deacetylase, enabling it to control the cellular activity of numerous acetylated proteins in mammals (Singh et al., 2019).

**TABLE 3:** Docking data on compounds.

Sr. no	Ligands	Aldose reductase (PDB ID: 1USO)	Tyrosine kinase (PDB ID: 1IR3)	^a^SIRT6 (PDB ID: 3K35)
1.	Luteolin	−12.3 kcal/mol	−8.6 kcal/mol	−8.9 kcal/mol
2.	Eriodictyol	−12.1 kcal/mol	−8.0 kcal/mol	−9.1 kcal/mol
3.	Astilbin	−11.5 kcal/mol	−8.2 kcal/mol	−9.0 kcal/mol
4.	Syringaresinol	−11.4 kcal/mol	−7.9 kcal/mol	−7.4 kcal/mol

^a^ SIRT6: sirtuin family of NAD(+)-dependent protein deacetylases.

**FIGURE 11:**
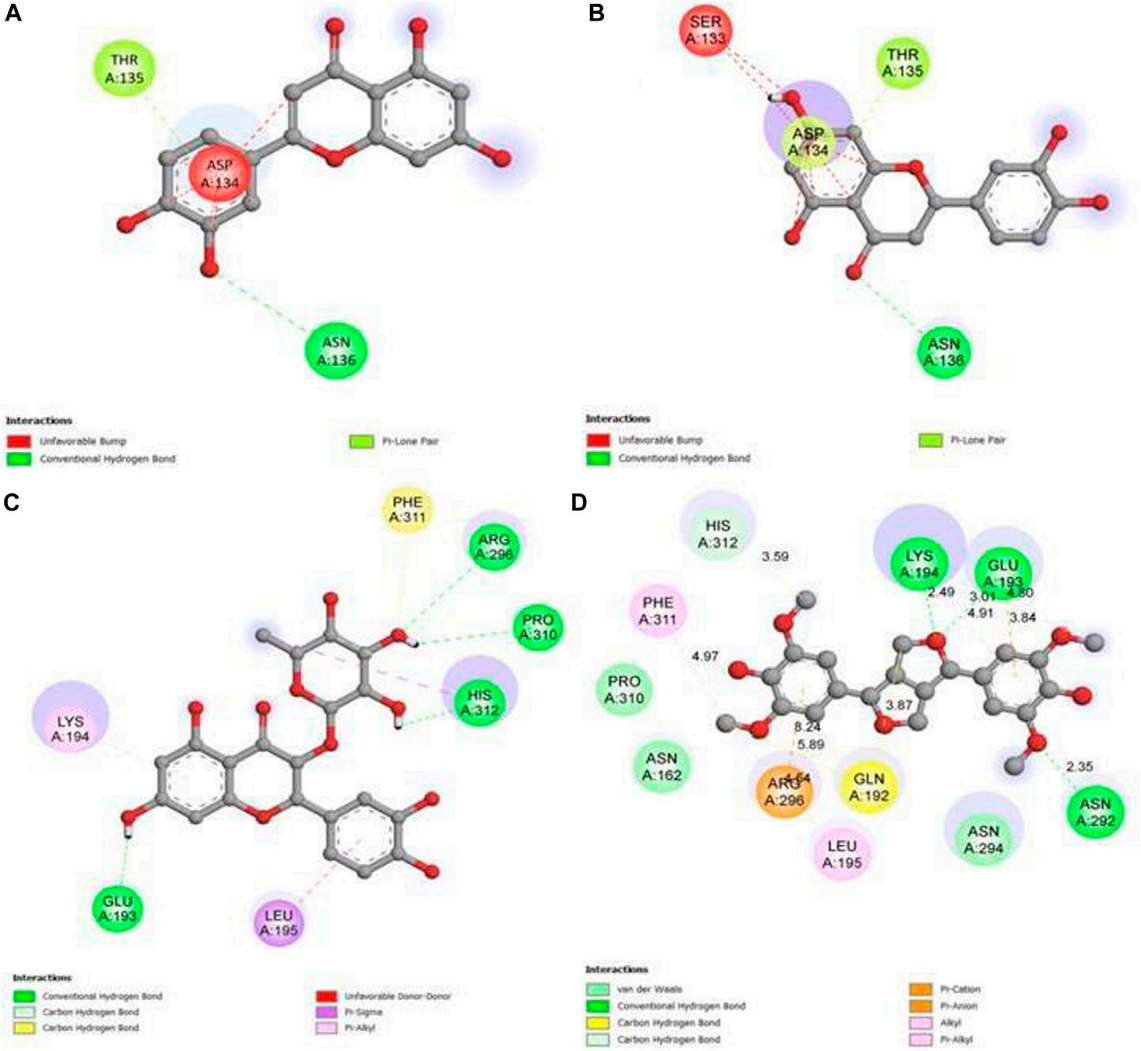
2D graph showing interactions at the molecular level for **(A)** pocket residues linked to luteolin, **(B)** pocket residues linked to eriodictyol, **(C)** pocket residues linked to astilbin, and **(D)** pocket residues linked to syringaresinol.

Our study revealed that luteolin (−12.3 kcal/mol) was most effective against the target receptor aldose reductase (PDB ID: 1USO), followed by eriodictyol (−12.1 kcal/mol), astilbin (−11.5 kcal/mol), and syringaresinol (11.4 kcal/mol). The molecular interaction of the binding pocket suggests that the bound ligand luteolin can potentially inhibit aldose reductase (PDB ID: 1USO); three key amino acid residues of AR (ASP134, THR135, and ASN136) were strongly forming hydrogen bonds, indicating strong binding to the active pocket (Figure 11A). Ligand eriodictyol can potentially inhibit aldose reductase (PDB ID: 1USO); four key amino acid residues of AR (SER133, ASP134, THR135, and ASN136) were strongly forming hydrogen bonds, suggesting strong binding to the active pocket (Figure 11B). Similarly, seven residues of AR (PDB ID: 1USO) protein (GLU193, LYS194, LEU195, ARG296, PRO310, PHE311, and HIS312) were strongly forming hydrogen bonds with astilbin, suggesting strong binding to the active pocket (Figure 11C). In the same way, ligand syringaresinol was involved in inhibiting the AR protein by binding with the 11 residues of AR protein (ASN162, GLN192, GLU193, LYS194, LEU195, ASN292,ASN294, ARG296, PRO310, PHE311, and HIS312) that were strongly forming hydrogen bonds, suggesting strong binding to the active pocket (Figure 11D). As a result, it may be concluded that luteolin is the most effective inhibitor of the AR protein and may act to improve glucose absorption as well as lowering hyperglycemia.

### ADMET analysis and drug-likeness

The molinspiration bioactivity scores were calculated as a measure of active drug-likeness of the molecules in terms of various parameters like kinase inhibitors, ion channel modulators, GPCR ligands, protease inhibitors, nuclear receptor ligands, and other enzyme inhibitors; these are presented in Table 4. In the luteolin study, higher bioactivity scores were observed for nuclear receptor ligands, enzyme inhibitors, and kinase inhibitors, with scores of 0.39, 0.28, and 0.26, respectively. In the eriodictyol study, higher bioactivity score were observed for GPCR ligands, nuclear receptor ligands, and enzyme inhibitors, with scores of 0.07, 0.46, and 0.21, respectively. In the astilbin study, higher bioactivity scores were observed for GPCR ligands, ion channel modulators, kinase inhibitors, nuclear receptor ligands, protease inhibitors, and enzyme inhibitors, with scores of 0.11, 0.05, 0.03, 0.12, 0.15, and 0.33, respectively. Finally, in the syringaresinol study, a higher bioactivity score of 0.08 was observed for enzyme inhibitors. The druglikeness data and pharmacokinetic properties are summarized in Table 5. According to the ADMET properties, all four effective binding compounds identified were found to be water-soluble; the literature reports that water solubility is an important property that influences absorption. All four compounds possess better solubility, which favors oral bioavailability. Luteolin, eriodictyol, and syringaresinol were predicted to have high intestinal absorption, but astilbin was not. Among these four compounds, astilbin was found to be the most skin-permeant, with a log Kp value of −9.15. Eriodictyol, astilbin, and syringaresinol were predicted to be P-gp substrates, while luteolin was not. P-gp is key member of the set of ATP-binding cassette transporters, which play a role in absorption of drugs by protecting cells. All four of the compounds were found to have lower blood–brain barrier (BBB) permeability, meaning that they were found to be poorly distributed in the brain. Luteolin, eriodictyol, and syringaresinol registered bioavailability scores of 0.55, whereas astilbin registered a bioavailability score of 0.17. This means that luteolin, eriodictyol, and syringaresinol passed the rule of five criterion, with better absorption, but astilbin failed to do so, with poor absorption.

**TABLE 4:** Prediction of bioactivity of ligands by molinspiration.

Ligands	GPCR ligand	Ion channel modulator	Kinase inhibitor	Nuclear receptor ligand	Protease inhibitor	Enzyme inhibitor
Luteolin	−0.02↓ ↓↓	−0.07↓ ↓↓	0.26↑↑↑	0.39↑↑↑	0.22↑↑↑	0.28↑↑↑
Eriodictyol	0.07 ↑↑↑	−0.20↓ ↓	−0.22↓ ↓↓	0.46↑↑↑	−0.09↓ ↓↓	0.21↑↑↑
Astilbin	0.11 ↑↑↑	0.05 ↑↑↑	0.03↑↑↑	0.12↑↑↑	0.15↑↑↑	0.33↑↑↑
Syringaresinol	−0.01↓ ↓↓	−0.23↓ ↓	−0.17↓ ↓↓	−0.01↓ ↓↓	−0.14↓ ↓↓	0.08↑↑↑

Downward arrows represent low bioactivity; upward arrows represent high bioactivity.

**TABLE 5:** *In silico* pharmacokinetics, physico-chemical and ADMET properties, and drug-likeness of ligands.

Sr. no	ADMET properties	Luteolin	Eriodictyol	Astilbin	Syringaresinol
1.	Molecular weight	286.24 g/mol	288.25 g/mol	450.39 g/mol	418.44 g/mol
2.	Num. heavy atoms	21	21	32	30
3.	Num. H-bond acceptors	6	6	11	8
4.	Num. H-bond donors	4	4	7	2
5.	Water solubility—ESOL class	Soluble	Soluble	Soluble	Soluble
6.	Intestinal absorption in humans	High	High	Low	High
7.	BBB permeability (log BB)	No	No	No	No
8.	P-gp substrate	No	Yes	Yes	Yes
9.	CYP1A2 inhibitor	Yes	No	No	No
10.	CYP2C19 inhibitor	No	No	No	No
11.	CYP2C9 inhibitor	No	No	No	No
12.	CYP2D6 inhibitor	Yes	No	No	Yes
13.	CYP3A4 inhibitor	Yes	Yes	No	No
14.	Log Kp (skin permeation)	−6.25 cm/s	−6.62 cm/s	−9.15 cm/s	−7.27 cm/s
15.	Lipinski	Yes	Yes	No (2 violations)	Yes
16.	Ghose	Yes	Yes	Yes	Yes
17.	Veber	Yes	Yes	No (1 violation)	Yes
18.	Egan	Yes	Yes	No (1 violation)	Yes
19.	Muegge	Yes	Yes	No (1 violation)	Yes
20.	Bioavailability score	0.55	0.55	0.17	0.55
21.	PAINS	1 alert	1 alert	1 alert	0 alerts
22.	Brenk	1 alert	1 alert	1 alert	0 alerts
23.	Leadlikeness	Yes	Yes	No (1 violation)	No (1 violation)
24.	Synthetic accessibility	3.02	3.11	5.27	4.36

### MD simulation of protein–ligand complexes

**FIGURE 12:**
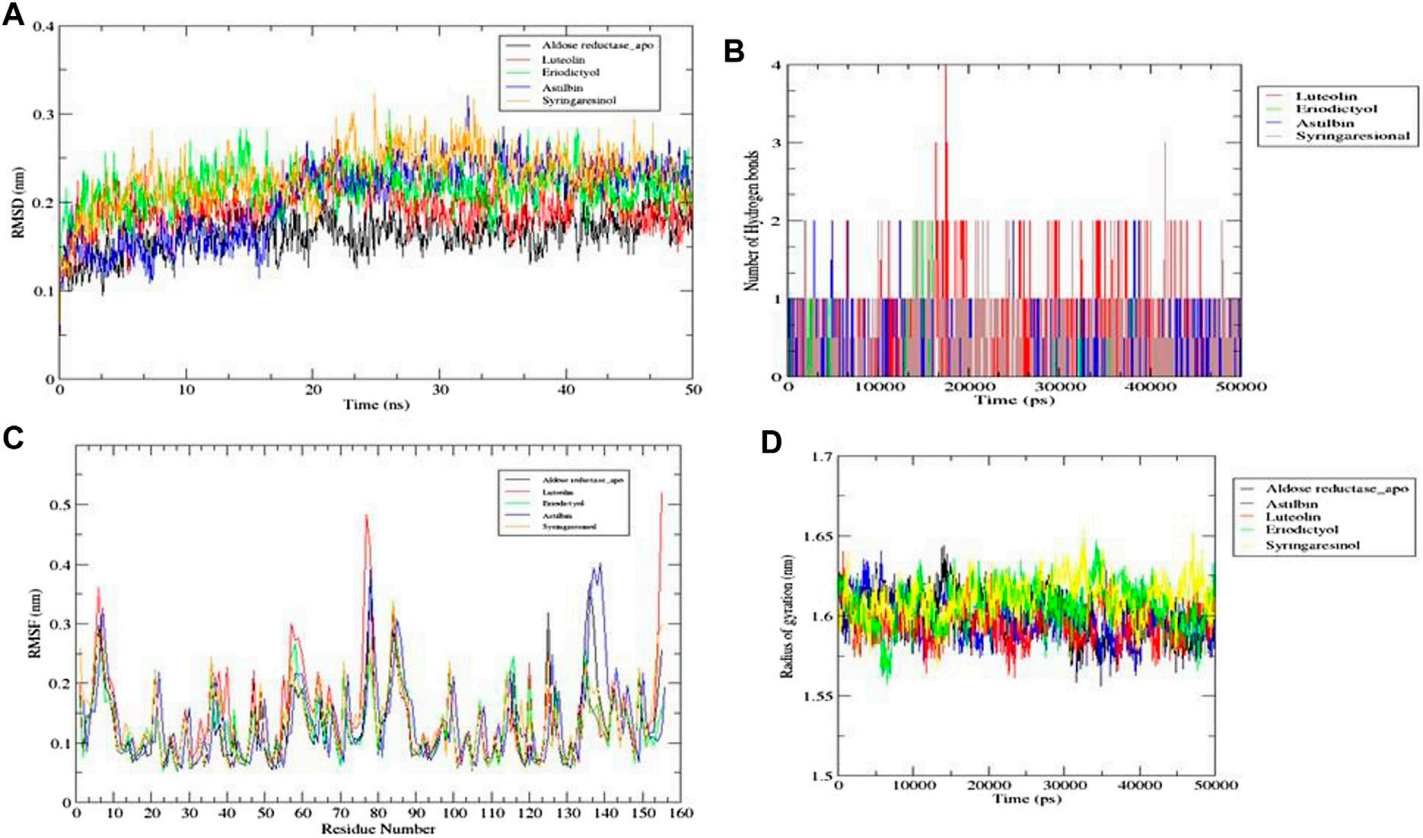
Molecular dynamics simulations. **(A)** RMSD graph for aldose reductase (AR) (PDB ID: 1USO) with luteolin, eriodictyol, astilbin, and syringaresinol for 50 ns. **(B)** RMSF graph for aldose reductase (AR) (PDB ID: 1USO) with luteolin, eriodictyol, astilbin, and syringaresinol for 50 ns. **(C)** Number of intermolecular hydrogen bonds between the ligands (luteolin, eriodictyol, astilbin, and syringaresinol) and amino acid residue of target protein aldose reductase (AR) (PDB ID: 1USO) for 50 ns. **(D)** Radius of gyration (RoG) results for luteolin, eriodictyol, astilbin, and syringaresinol with aldose reductase (AR) for 50 ns.

Of the ten ligands that were screened against the three receptor targets, luteolin, eriodictyol, astilbin, and syringaresinol showed the best binding affinity with aldose reductase (AR) (PDB ID: 1USO). Although all four ligands followed all molecular dynamics properties such as conformational stability, flexibility, and compactness of protein-ligand complexes measured by (RMSD, RMSF, RoG, and intermolecular hydrogen binding) in the MD simulations, luteolin showed the most promising potential, because it was constant throughout the 50 ns simulation period (Figures 12A, B). Luteolin showed the maximum number of hydrogen bonds and a high RoG value compared to the other compounds at the end of the 50 ns simulation (Figures 12C, D).

## Discussion

Flavonoids are the largest group of polyphenols and are well known as AGE inhibitors of BSA glycation (Yeh et al., 2017). It is also evident that flavonoids have antioxidant and antiglycation properties which might be associated with their anti-oxidation function (Anusiri et al., 2014). *Lyonia ovalifolia* has previously been reported to exhibit antioxidant, antimicrobial, and immuno-modulatory effects (Acharya, 2015), and to show cytotoxic and antidiabetic activity (Yang et al., 2017). In this study, the effects of the ethanolic extract and solvent fractions of *L. ovalifolia* on AGE production were found to indicate that ethanolic extract has considerable potential to inhibit AGE formation at both early and advanced stages of glycation. Moreover, at 100 μg/mL, the L-03 bioactive fraction showed better AGE inhibition in the early phases of glycation compared to AG (positive control).

Similarly, L-01 was found to stimulate the translocation of GLUT4 molecules at the cell surface, dose dependently, and alone as well as in combination with insulin. This suggests that L-01 increases GLUT4 translocation in L6 GLUT4 myc skeletal muscle cells and has potential implications for the management of Type 2 diabetes. These findings further validate our discovery that L-01 follows a similar pathway to insulin for the stimulation of GLUT4 translocation to the cell surface. A similar finding regarding the antidiabetic potential of *L. ovalifolia* has been reported recently, in which eriodictyol isolated from ethanolic extract was found to stimulate insulin secretion though cAMP/PKA signaling (Hameed et al., 2018). Moreover, three new iridoid compounds have been isolated that also showed antihyperglycemic effects (Hussain et al., 2018). Cytochrome (CYP450) is well known for drug metabolism; here, we observed that isomers of CYP450 are not affected by any of the four compounds identified, meaning that compounds do not suppress the metabolic functions of involved enzymes. Regarding log Kp values, the more negative the value, the lower the availability of a compound to pass through the skin (Hussain et al., 2021). The bioavailability scores of luteolin, eriodictyol, and syringaresinol suggested that they follow the Lipinski, Veber, Ghose, Egan, and Muegge rule.

The antiglycation and GLUT4 translocation potential of *L. ovalifolia* is supported by the presence of flavonoids in the LCMS determinants of the L-03 fraction, namely, eriodictyol, quercitrin (quercetin-3-O-rhamnoside), quercetin-3-O-galactoside, luteolin, and astilbin (dihydroquercetin-3-O-rhamnoside). The docking studies also supported the hypothesis of GLUT4 translocation potential. Various reports support the flavonoids and their antidiabetic activity (Yasue and Kato, 1959; Yasue et al., 1961; Rahman et al., 2007; Dong et al., 2017; Hameed et al., 2018; Ma et al., 2021). There are several pieces of evidence linking the intake of flavonoids with favorable outcomes among patients with Type 2 diabetes (Wu and Yen, 2005; Wedick et al., 2012). The literature also reveals that flavonoids from the leaf extract of *Thuja orientalis*; their isolated flavonoids (i.e., quercitrin and amentoflavone) inhibit aldose reductase as well as the formation of AGEs and exhibit the strongest potential for AGE inhibition (Lee et al., 2009). Three flavonoids isolated from *Cephalotaxus sinensis* leaves have also been found to show an association with elevated GLUT4 protein levels in diabetic rats (Li et al., 2007). The experimental evidence reported in this article suggests that *L. ovalifolia* is a rich source of naturally occurring flavonoids. These compounds showed strong inhibitory potential against AGE formation and, hence, antiglycation activity, along with enhanced GLUT4 activity; this finding was also supported by docking studies.

## Conclusion

This study shows that crude extract of *L. ovalifolia* has the potential to perform dual activities: antiglycation of advanced glycated endproducts (AGEs) as well as GLUT4 translocation. The strong antiglycation activity of the ethyl acetate fraction might be due to the involvement of flavonoids. The reported evidence strongly supports the anti-diabetic properties of *L. ovalifolia*, which may be considered to be a new and renewable herbal source for the management of insulin resistance and long-term diabetic complications. The promising potential of all four compounds, as evaluated in docking studies, MD simulations, and ADMET analysis, recommends them for *in vitro* investigation as well as *in vivo* experimentation to confirm their value in drug development.

## Data Availability

The datasets presented in this study can be found in online repositories. The names of the repository/repositories and accession number(s) can be found in the article/supplementary material.
